# Anti-depressant effects of oil from fructus gardeniae via PKA-CREB-BDNF signaling

**DOI:** 10.1042/BSR20190141

**Published:** 2019-04-26

**Authors:** Jie Ruan, Li Liu, Xin Shan, Baomei Xia, Qiang Fu

**Affiliations:** 1 Department of Pharmacology of Chinese Materia Medica, China Pharmaceutical University, Nanjing 211198, China; 2 Guangdong Medical University, Dongguan 523808, China

**Keywords:** Depression, Gardenia jasminoides oil extract, PKA/CREB/BDNF

## Abstract

The dried ripe fruit of Gardenia jasminoides Ellis was usually applied as an herb medicine in Traditional Chinese Medicine. It was suggested that the Gardenia jasminoides oil extract (oil from Fructus Gardeniae [OFG]) might serve as a potential treatment for depression, whereas its pathogenesis still remained not fully understood. The present research was conducted to evaluate the anti-depressive effect of OFG in mice and explore its potential mechanism. The OFG and ketamine (KET) were intragastrically and intraperitoneally treated, respectively. Thereafter, the animals were subjected to the behavior tests. The expressions of protein kinase A (PKA), brain derived neurotrophic factor (BDNF), cAMP response element-binding protein (CREB) in hippocampus were detected by Western blot. The selective PKA inhibitor H-89 was also applied to confirm the mechanism. As a result, OFG and KET treatment improved the behavior performance. Furthermore, the administrations of OFG effectively enhanced the expressions of PKA, p-CREB, and BDNF. With the application of selective PKA inhibitor H-89, the ameliorated effects caused by OFG were blocked, but not by KET. In conclusion, the presented work indicated that OFG-exerted protective effect on depression through PKA-CREB-BDNF signaling.

## Instruction

Depression and stress-related mental disorder lead to significant personal, economic, and social burdens [1–3]. Depression is a common major depressive disorder (MDD) with pathological changes in brain. The hallmarks of MDD are anorexia, hypothermia, pessimism, sustained low mood, and suicide tendency, which cause great pain on individuals and their families [3]. According to the World Health Organization report in 2012, 350 million people suffered from depression, and depression would become the second leading disease by 2020 [4]. However, some depressive patients are resistant to current available antidepressants in the clinical intervention. In addition, a large number of clinical reports indicated that several antidepressants showed partially reactive or unresponsive therapeutic activity with severe side effects [5]. Therefore, it is urgent to search for new medications of depression.

The dried ripe fruit of Gardenia jasminoides Ellis is usually applied as a herb medicine in Traditional Chinese Medicine and has been recently acknowledged as Fructus Gardeniae (FG) in Chinese Pharmacopoeia. FG is reported to possess diverse properties including hepatoprotective, anti-oxidative, anti-inflammatory, anti-depressive, anxiolytic, and anti-psychotic effects [6–8]. Among the several effective chemical constitutes, the water soluble compounds including iridoid glycosides and crocins were considered to be the potential anti-depressants. Studies have shown that the non-polar extract of Gardenia Jasminoides has a rapid anti-depressant activity, which mainly activated by the brain-derived neurotrophic factor (BDNF)-tropomyosin-related kinase receptor B (TrkB) cascade [9]. It was illustrated that the water extract of the fixed combination containing Gardenia jasminoides exerted anti-depressant property [10]. Our previous work also elicited that non-polar oil from Fructus Gardeniae (OFG) might serve as a potential treatment for depression, without the detection of underlying mechanism [11]. However, the pathogenesis of OFG-related anti-depressive effect still remained not fully understood.

BDNF expression is regulated by a variety of signaling pathways including cAMP response element binding (CREB), which is one of the most studied transcription factors involved in the etiology of depressive and anti-depressive responses. Human post-mortem study reported a decrease in hippocampal CREB expression in patients with MDD. [12] Previous research proposed a down-regulation of CREB activity in chronic stress models in rodents. [13] Long-term treatment of fluoxetine enhances cAMP levels, subsequently activates protein kinase A (PKA) and up-regulates CREB mRNA in chronic stress-stimulated hippocampus, cortex and hypothalamus [14]. CREB signaling regulates the expression of genes which promote synapses and neural plasticity, as evidenced by the presence of CREB elements in the BDNF promoter region [15–17].

CREB, a kind of regulatory factor in nuclei, is an important event of multiple intracellular signaling pathways in the nervous system [18,19]. CREB signaling in the hippocampus has been implicated in the impairment of psychotic and cognitive behaviors. It was elicited that PKA was required for the activation of CREB. Previous studies demonstrated that stress-dependent differences in CREB signaling contributed to the alterations of learning and memory-related behaviors [20–24]. BDNF is one of the major downstream target genes of CREB, which is the most prevalent neurotropic factor in the cerebral dysfunction. BDNF is also acknowledged as one of the most extensively investigated target molecules controlling brain plasticity, survival, and differentiation in both periphery and central nervous system (CNS) [25]. Studies have shown that the non-polar extract of Gardenia Jasminoides has a rapid antidepressant activity, which mainly depends on the BDNF-TrkB signaling [9]. In our previous study, oil component prepared from the Fruit of Gardenia jasminoides (OFG) using the supercritical fluid carbon dioxide extraction might contain effective constituent which could be used for depression therapy [11]. Therefore, it was assumed that OFG functioned as an anti-depressant possibly based on the expression of BDNF and CREB. Herein, the present study was aimed to evaluate the anti-depressant activity of OFG and explain its correlated mechanism.

## Results

### The effects of OFG on the immobile duration in tail suspension test and forced swim test

The immobility time of tail suspension test (TST) and forced swim test (FST) was measured to evaluate the effect of OFG on depressive-like behavior. The mice received different dosage of OFG displayed significant reductions in immobility time compared with those in control group during the TST and FST (Figure 2A) (One-way analysis of variance (ANOVA), TST: F (4, 44) = 6.32, *P*<0.001; FST: F(4, 45) = 14.42). The ketamine (KET) administration also presented inhibitory effects on immobile duration in TST and FST.

### Effects of OFG on the distance traveled and the center time in open field test

The spontaneous locomotor activity was estimated in open field test (OFT). As illustrated in Figure 3, the exposure to OFG (0.48g/kg) and KET (30 mg/kg) showed no statistical influence on the distance traveled (ANOVA, F (2, 21) = 1.821, *P*=0.186). Besides, the administrations of OFG and KET scarcely affected the total time spent in central region (ANOVA, F (2, 21) = 1.902, *P*=0.174). Thus, it could be excluded that the anti-depressive like behavior was due to the dysfunction of locomotor activity.

### Effects of OFG on the latency to feed and food consumptions in the novelty-suppressed feeding

Next, we examined the novelty-suppressed feeding (NSF) paradigms test to further evaluate the anti-depressive effect of OFG. As depicted in Figure 4, the OFG treatment produced an anti-anxiety response in the NSF test. In brief, the administrations of OFG and KET decreased the latency to begin feeding as compared with that in control group (ANOVA, F (2, 23) = 29.17, *P*<0.01) (Figure 4A). While the mice treated with OFG or KET also significantly consumed more food than control animals (ANOVA, F (2, 23) = 15.56, *P*<0.01) (Figure 4b).

### Effects of OFG on the hippocampal PKA, CREB, and BDNF protein levels after 24 h

The protein expressions of PKA, CREB, and BDNF in hippocampus were detected for the underlying mechanism of OFG-mediated depression. As revealed in Figure 5, the expressions of PKA and BDNF in OFG-treated group were obviously up-regulated compared with those in control group (*P*<0.05), which were less potent than those in KET-treated group (*P*<0.01) (ANOVA, PKA: F (2, 15) = 10.37, *P*<0.01; BDNF: F (2, 15) = 7.614, *P*<0.01). Moreover, the expression of p-CREB was evidently enhanced compared with that in control group (*P*<0.01), which was more efficient than that in KET group (ANOVA, F (2, 15) = 11.55, *P*<0.01).

### Blockade of PKA-CREB signaling blunted antidepressant effects and up-regulation of BDNF expression by OFG, but not KET

To examine the underlying pathogenesis, we treated the mice with PKA selective inhibitor H-89 and estimated the immobility duration in FST using Two-way ANOVA analysis. As presented in Figure 6, with the intervention of H-89, the immobility time of FST was notably increased (*P*<0.01) in OFG-treated group than that in OFG-treated group without H-89 inhibition. It was also showed that the H-89 plus KET group presented no significant effect than KET-treated group without H-89 inhibition (*P*=0.99). Besides, the H-89 group presented no significant difference than that in vehicle group.

We also detected the effect of H-89 on the expressions of p-CREB and BDNF. H-89 pre-treatment also inhibited up-regulation of BDNF expression induced by OFG. A two-way ANOVA revealed significant differences for the H-89 pre-treatment (F (1, 20) = 6.33, *P*<0.05), OFG treatment (F (1, 20) = 12.62, *P*<0.01), and H-89 × OFG interaction (F (1, 20) = 8.635, *P*<0.01, Figure 6B). In contrast, H-89 could not reverse up-regulation of BDNF expression induced by KET: a two-way ANOVA revealed the significant effect of KET treatment (F (1, 20) = 56.42, *P*<0.01), but not H-89 pre-treatment (F (1, 20) = 0.079, *P*=0.781) or H-89 × KET interaction (F (1, 20) = 0.001, *P*=0.989).

In addition, H-89 pre-treatment also suppressed the up-regulation of CREB expression caused by OFG. A two-way ANOVA revealed significant differences for the H-89 pre-treatment [F (1, 20) = 4.763, *P*<0.05], OFG treatment (F(1, 20) = 8.467, *P*<0.01) and H-89 × OFG ((1, 20) = 8.402, *P*<0.01, Figure 4C). Meanwhile, neither KET nor H-89 affected the CREB level (two way ANOVA, F (1, 20) = 0.068, *P*=0.796 for KET treatment; H-89: F (1, 20) = 0.001, *P*=0.992 for H-89 pre-treatment). These results suggested that the anti-depressant effect of OFG, but not KET, was dependent on the PKA/CREB/BDNF pathway.

## Discussion

In previous studies, it was found that the administration of OFG was effective in relieving the behavioral despair in normal mice [11]. Thus, it was hypothesized that OFG might exert beneficial effect on depressive-like behavior. Therefore, we investigated the anti-depressant like effect of OFG and explored its potential mechanism with murine depressive model. Compared with the control group, it was found that the treatment of OFG could significantly reduce the immobility duration in the FST and TST. We further demonstrated that the administrations of OFG or KET significantly reduced depressive-like symptoms by decreasing total ambulation, central ambulation, and feeding latency in comparison with that of control mice [26], which confirmed the anti-depressive like property of OFG and KET.

CREB is an important component of multiple intracellular signaling pathways in the nervous system and is capable of modulating transcription by autophosphorylation. Various intracellular signal transduction cascades are proposed directly or indirectly to mediate the activation of CREB. Several enzymes, such as PKA, protein kinase C (PKC), Ca^2+^/calmodulin-dependent protein kinase (CAMKII), extracellular-regulated protein kinase (ERK), phosphoinositide 3-kinase (PI3K), and glycogen synthase kinase 3 (GSK-3), are suggested to participate in the promotion of CREB [27,28]. CREB signaling in the hippocampus has been implicated in emotional and cognitive behaviors. Furthermore, CREB is implicated in the transcriptional regulation of both stress and depression. The phosphorylation of CREB is governed by cAMP PKA, which has been elicited to control various psychiatric disease including major depression [29]. As one of the downstream target of PKA/CREB signaling, neurotropic factor BDNF participates in most multiple brain dysfunctions. Furthermore, a recent study has demonstrated that in the cultured neurons, BDNF initiates self-amplification of BDNF mRNA expression through various signaling pathways including PKA-CREB cascade [23]. BDNF can be recruited by CREB phosphorylation and is responsible for neuronal function. Several studies displayed that BDNF down-regulation contributed to structural damage and functional impairment in the CNS, which was associated with depressive symptoms both in mice and humans. Recent investigation has shown that BDNF represses depressive symptoms mainly by binding to TrkB, and leading to autophosphorylation of TrkB tyrosine residues [30]. After binding with and activating TrkB, BDNF is regarded to suppress the pathophysiology of depression.

KET, the NMDA receptor antagonist for glutamate, has been widely applied for the investigation of anti-depressants.

BDNF-associated signaling is involved in the mechanism of rapid anti-depressant effect of KET [31]. Liu et al. [32] elicited that the anti-depressant effects of KET in chronic unpredictable stress depressive model was attributed to BDNF-TrkB signaling. It was displayed that the co-treatment with KET induced antidepressant-like behavior through the up-regulation of PKA, BDNF, and CREB [29]. In addition, the ethanol extract of Gardenia jasminoides Ellis showed rapid anti-depressant-effect by upr-egulating BDNF expression [33]. The Gardenia yellow pigment (GYP), enriched in Gardenia jasminoides Ellis, was also confirmed to exert anti-depressant activity by promoting CREB and BDNF [34]. The above evidence indicated that KET and OFG might exhibit anti-depressive like behavior through PKA/CREB/BDNF pathway.

Herein, we demonstrated the expressions of p-CREB and BDNF were evidently up-regulated in hippocampus at 24  h post-OFG administration. The increased p-CREB expression was possibly attributed to the up-regulation of total CREB and PKA protein expressions. After the combination and activation of TrkB, BDNF was thought to underlie OFG for the treatment of depression. PKA signaling plays a critical role in the modulation of cerebral functions, such as neurotransmitter release and post-synaptic responses to neurotransmitters, which are closely related to the intervention of depressive-like behavior. Former literature showed that the regulation of PKA/CREB/BDNF signaling pathways could achieve anti-depressant effects [35]. Thus, H-89, the selective inhibitor of PKA, was applied to verify the critical role of PKA in the mediation of OFG-regulated anti-depressive property. The data illustrated that the suppression of PKA markedly abolished the augment of immobile duration in FST, and the over-expressions of p-CREB and BDNF. It was confirmed that the anti-depressant like activity of OFG was due to the regulation of PKA/CREB/BDNF signaling pathway. These data strongly highlighted the anti-depressant like effects of OFG in mice.

In conclusion, the present study demonstrated that OFG exhibited anti-depressive like effect possibly through the PKA/CREB/BDNF signaling. Further investigations are warranted with the transgenetic animals to confirm the crucial role of PKA during this pathogenesis.

## Method

### Animals

Male 7–8 weeks Kunming (KM) mice were obtained from Academy of Military Medical Sciences. The mice were housed in the standard condition at constant temperature-controlled (22–25°C) room with a 12-h light/dark cycle. The animals had free access to standard food and water *ad libitum*. All animal procedures were carried out in accordance with the Guide for the Care and Use of Laboratory Animals approved by the Institutional Animal Care and Use Committee at Nanjing University of Chinese medicine (permission number IACUC-20160613).

### Drugs and treatment

OFG was conducted as described in our previous investigation [21]. The mice were randomly assigned to four groups: control group, OFG (0.24 g/kg) group, OFG (0.48 g/kg) group, and OFG (0.96 g/kg) group, and KET (30 mg/kg) group. The OFG and KET (30 mg/kg), dissolved in 0.5% (w/v) Tween 80, were intragastrically and intraperitoneally administered, respectively. Simultaneously, the control groups received the same volume of vehicle. The dosage of KET was chosen according to our previous research [36]. Thereafter, the animals were subjected to TST and FST. As OFG (0.48 g/kg) group showed slightly more efficacy on reducing immobile duration than OFG (0.24 g/kg) group and OFG (0.96 g/kg) group, we chose control group, OFG (0.48 g/kg) and KET group for the future tests. The FST was carried out 6 h after TST, and NSF was carried out 6 h after OFT.

Additionally, in order to explore the role of PKA/CREB/BDNF signaling in the OFG-mediated anti-depressant effect, new male 7–8 weeks Kunming (KM) mice were purchased and randomly divided to vehicle + saline group, OFG (0.48 g/kg) + saline group, KET (30 mg/kg) + saline group, vehicle + H-89 group, OFG (0.48 g/kg) + H-89 group, KET (30 mg/kg) + H-89 group. The treatments with OFG and KET were carried out in accordance with the above description. H-89 at 10 mg/kg (Sigma, St. Louis, MO, U.S.A.) or normal saline were dissolved in 0.5% DMSO (dimethyl sulfoxide) and intraperitoneally injected 30 min before drug administrations. The 2 days later, FST was conducted.

**Figure 1:**
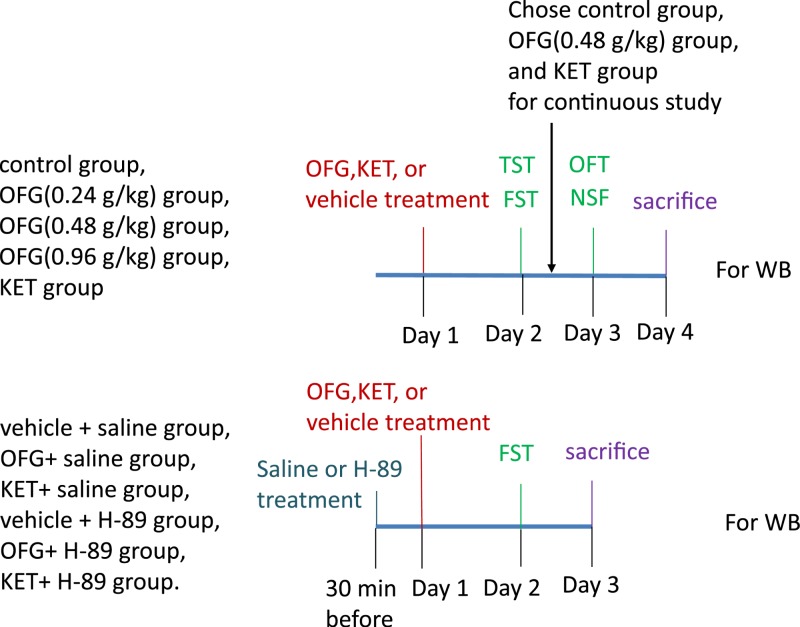
The procedure description of the present study

After the behavior tests, the mice were killed by decapitation. The brain tissues were quickly harvested and stored at −80°C for pending test. The procedure description was illustrated in Figure 1.

**Figure 2:**
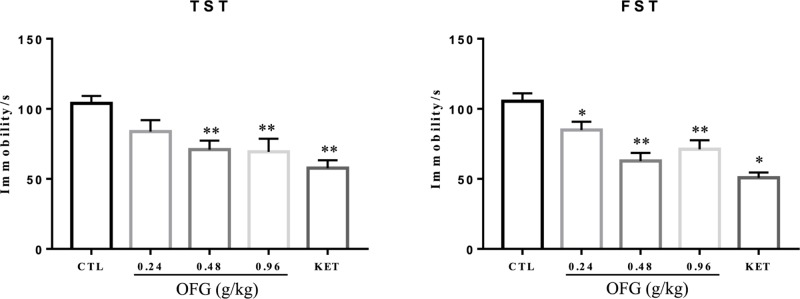
Effects of OFG on the TST and FST Immobility time was measured at 24-h post a single drug administration. Group: control, CTL (saline), OFG (0.24 g/kg, 0.48 g/kg, 0.96 g/kg), KET (30 mg/kg). One-way ANOVA, TST: F (4, 44) = 6.32, *P*<0.001, *n*=9–10/group; FST: F(4, 45) = 14.42, *P*<0.001, *n*=10/group. ^*^P<0.05, ^**^P<0.01, vs control. Data were expressed as mean ± S.E.M.

**Figure 3:**
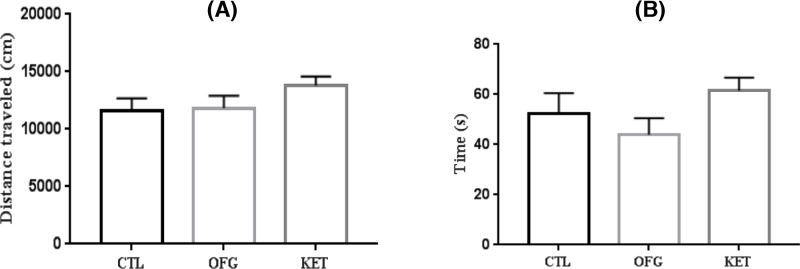
Effects of OFG on the OFT at 24-h post-CTL (saline), OFG (0.48 g/kg), Ket (30 mg/kg) administration (**A**) Total distance traveled during a 5-min open field testing time, ANOVA, F (2, 21) = 1.821, *P*=0.186), *n*=8–10/group; (**B**) Time spent on the center part during a 5-min open field testing time. ANOVA, F (2, 21) = 1.902, *P*=0.174. *n*=8–10/group, Data were expressed as means ± S.E.M.

**Figure 4:**
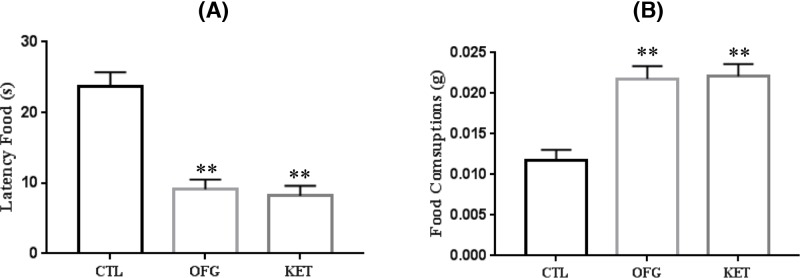
OFG produced an anti-depressive like response in NSF paradigms (**A**) OFG, KET both significantly decreased the time of latency to eat during 10-min test of NSF, ANOVA, F (2, 23) = 29.17, *P*<0.01, *n*=8–10/group; (**B**) There was a trend for the change of the total amount of food consumed, F (2,23) = 15.56, *P*<0.01. ^**^P<0.01, vs control. *n*=8–10/group, Data were expressed as mean ± S.E.M.

**Figure 5:**
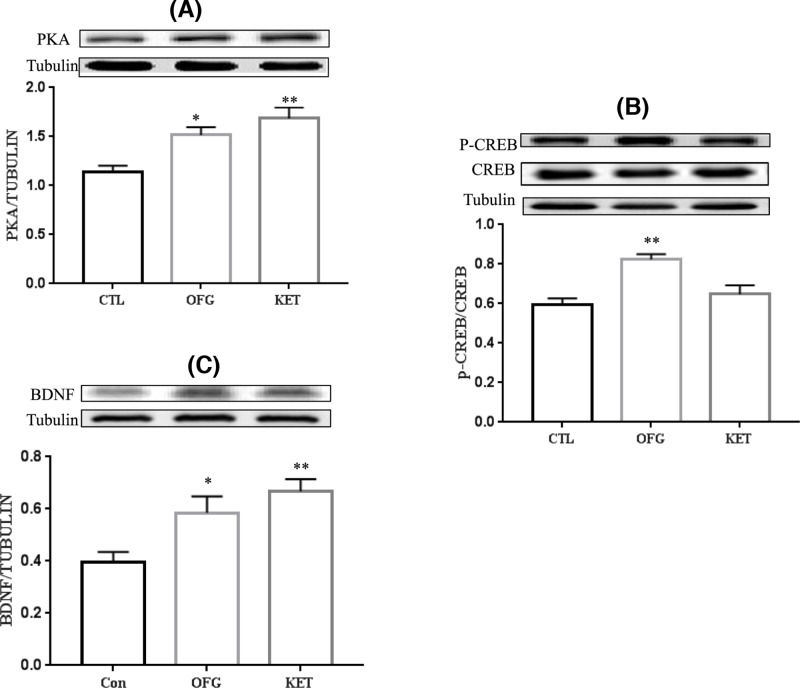
PKA/CREB/BDNF protein expression in the hippocampus after a single OFG/KET treatment (**A**) PKA expression significantly increased after a single administration of OFG and KET for 24 h, ANOVA, F (2, 15) = 10.37, *P*<0.01. ^*^P<0.05, ^**^P<0.01, vs control, *n*=6/group, Data represent mean ± S.E.M. (**B**) p-CREB/CREB expression significantly increased after a single administration of OFG for 24 h, ANOVA, F (2, 15) = 11.55, *P*<0.01. ^**^P<0.01, vs control, *n*=6/group, Data represent mean ± S.E.M. (**C**) BDNF expression significantly increased after a single administration of OFG and KET for 24 h, ANOVA, F (2, 15) = 7.614, *P*<0.01. ^*^P<0.05, ^**^P<0.01, vs control. *n*=6/group, Data were expressed as mean ± S.E.M**.**

**Figure 6:**
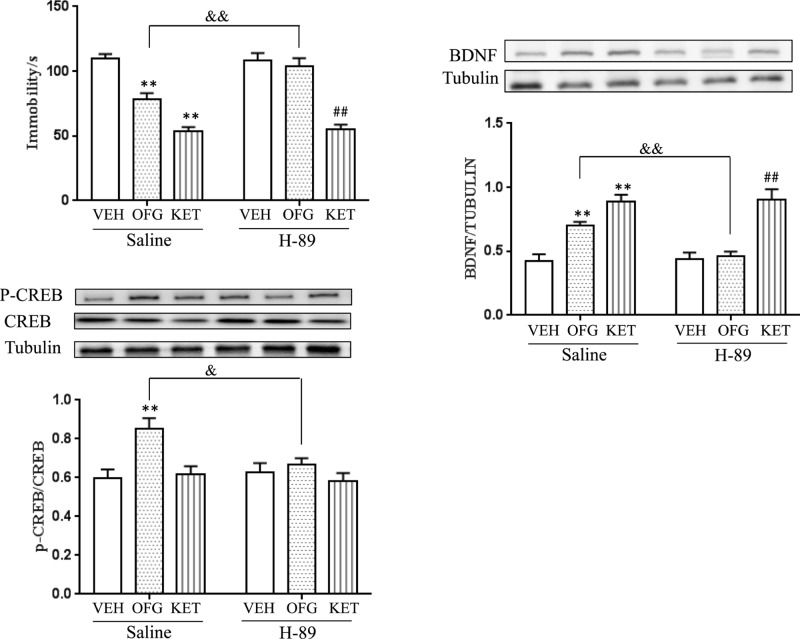
The effect of a PKA antagonist on antidepressant response and CREB/BDNF protein expression in the hippocampus after a single OFG/KET treatment (**A**) H-89 blocked the antidepressant response of OFG (F (1, 32) = 6.221, *P*<0.05); in contrast, there was no effect on KET (F (1, 32) = 0.007, *P*=0.932). *n*=9/group. ^**^P<0.01, compared with vehicle (VEH, saline); ^##^*P*<0.01, compared with vehicle (H-89); ^&&^*P*<0.01, compared with OFG. (**B**) H-89 reversed up-regulation of BDNF expression induced by OFG (F (1, 20) = 6.33, *P*<0.05); in contrast, there was no effect on KET (F (1, 20) = 0.08, *P*=0.781). *n*=6/group. ^**^P<0.01, compared with vehicle (saline); ^##^*P*<0.01, compared with vehicle (H-89); ^&^*P*<0.05, compared with OFG. (**C**) H-89 reversed up-regulation of p-CREB/CREB expression induced by OFG (F (1, 20) = 4.763, p < 0.05); in contrast, there was no effect on KET (F (1, 20) = 0.001, *P* = 0.993). *n*=6/group. ^**^P<0.01, compared with vehicle (saline); ^&^*P*<0.05, compared with OFG. Data were expressed as means ± S.E.M.

### Behavioral testing

#### Tail suspension test

The process for TST was performed acoustically and visually isolated using a computerized device with four individual detective equipment. Each mouse was suspended 0.5 m above the floor by adhesive tape at 1 cm from the top of the tail in a chamber. The sessions of the animals were videotaped. Data were calculated for the total duration of immobility in the final 4 min of the 6-min test [37]. Immobility was identified as a failure to make any struggling movements.

#### Forced swim test

The FST was carried out according to the previous research with minor modification [38]. Each mouse was individually forced to swim in an open cylindrical container (diameter 10 cm, height 25 cm), containing 15 cm of water at the constant temperature of 25 ± 1°C. The behavior of the individual mouse was videotaped. Data were counted for immobility period only in the last 4 min of the total test. The mice were considered immobile when they remained floating motionless in the water with only slight necessary movements to keep their nose above water.

#### Open field test

To assess locomotor activity and anxiety-like behavior, OFT was performed in a well-illuminated (∼300 lux) transparent acrylic area (40× 40× 15 cm). The mice were gently placed in peripheral zone of arena facing the wall and allowed to explore for 5 min. The camera was used to track the digitized image of the moving path. ANY-maze software was used to analyze the total running distance (locomotor activity) and the time spent in center. All experimenter should be kept out of the animal’s sight. The 70% ethanol was used to clean the testing apparatus during the interval of individual test.

#### Novelty-suppressed feeding test

The NSF was designed to evaluate the anxiolytic and/or antidepressant effects of chronic antidepressant treatment in rodents using a plastic box (50*50*20cm) [39]. At 18 h prior to behavioral testing, all food was removed from the rodent’s home cage. For the NSF test, a single weighed pellet of food was put in middle position of the plastic box on a white paper plate. Each mouse was placed in a corner of the box and allowed to explore for continuous 10 min. The trial was stopped when the mice had a bite on the chow. The amount of food consumed in the home cage was regarded as the weight of chow consumed in 10 min. The latency was recorded at the first time of food consuming.

### Western blot

The whole hippocampus was lysed in RIPA buffer containing protease and/or phosphatase inhibitors. Protein concentration was determined using commercial BCA assay (Pierce, Rockford, IL, U.S.A.). The protein lysates were separated by SDS/PAGE electrophoresis and were transferred onto polyvinylidene difluoride (PVDF) membranes. Antibodies for Western blots were supplied from Cell Signaling Technology and Abcam. After 1-h blockade with 5% BSA, the membranes were incubated with BDNF, TrkB, PKA, CREB, p-CREB, and β-Tubulin antibodies at 4°C overnight. Then the blots were incubated with suitable horseradish peroxidase-conjugated secondary antibody for 1 h. Consequently, the blots were visualized using the SuperSignal West Pico Chemiluminescent Substrate (Thermo Fisher Scientific Inc.). The immunoreactivities of BDNF, TrkB, PKA, p-CREB, and CREB were modified to β-Tubulin bands. All experiments were performed in triplicate.

### Statistical analyses

The statistical analyses were conducted by one-way ANOVA or two-way ANOVA by Graphpad 5.0, followed by a Bonferroni *post hoc* analysis if appropriate. The *P*-values less than 0.05 were considered to be statistically significant.

## Abbreviations

BDNF: brain-derived neurotrophic factor
cAMP: Cyclic Adenosine monophosphate
CREB: cAMP response element-binding protein
FST: forced swim test
KET: ketamine
MDD: major depressive disorder
NSF: novelty suppressed feeding
OFG: oil from Fructus Gardeniae
PKA: protein kinase A
PKC: protein kinase C
RIPA: Radio Immunoprecipitation Assay
TST: tail suspension test
